# ContourTL-Net: Contour-Based Transfer Learning Algorithm for Early-Stage Brain Tumor Detection

**DOI:** 10.1155/2024/6347920

**Published:** 2024-04-29

**Authors:** N. I. Md. Ashafuddula, Rafiqul Islam

**Affiliations:** Department of Computer Science and Engineering, Dhaka University of Engineering & Technology, Gazipur 1707, Bangladesh

## Abstract

Brain tumors are critical neurological ailments caused by uncontrolled cell growth in the brain or skull, often leading to death. An increasing patient longevity rate requires prompt detection; however, the complexities of brain tissue make early diagnosis challenging. Hence, automated tools are necessary to aid healthcare professionals. This study is particularly aimed at improving the efficacy of computerized brain tumor detection in a clinical setting through a deep learning model. Hence, a novel thresholding-based MRI image segmentation approach with a transfer learning model based on contour (ContourTL-Net) is suggested to facilitate the clinical detection of brain malignancies at an initial phase. The model utilizes contour-based analysis, which is critical for object detection, precise segmentation, and capturing subtle variations in tumor morphology. The model employs a VGG-16 architecture priorly trained on the “ImageNet” collection for feature extraction and categorization. The model is designed to utilize its ten nontrainable and three trainable convolutional layers and three dropout layers. The proposed ContourTL-Net model is evaluated on two benchmark datasets in four ways, among which an unseen case is considered as the clinical aspect. Validating a deep learning model on unseen data is crucial to determine the model's generalization capability, domain adaptation, robustness, and real-world applicability. Here, the presented model's outcomes demonstrate a highly accurate classification of the unseen data, achieving a perfect sensitivity and negative predictive value (NPV) of 100%, 98.60% specificity, 99.12% precision, 99.56% *F*1-score, and 99.46% accuracy. Additionally, the outcomes of the suggested model are compared with state-of-the-art methodologies to further enhance its effectiveness. The proposed solution outperforms the existing solutions in both seen and unseen data, with the potential to significantly improve brain tumor detection efficiency and accuracy, leading to earlier diagnoses and improved patient outcomes.

## 1. Introduction

**Figure 1:**
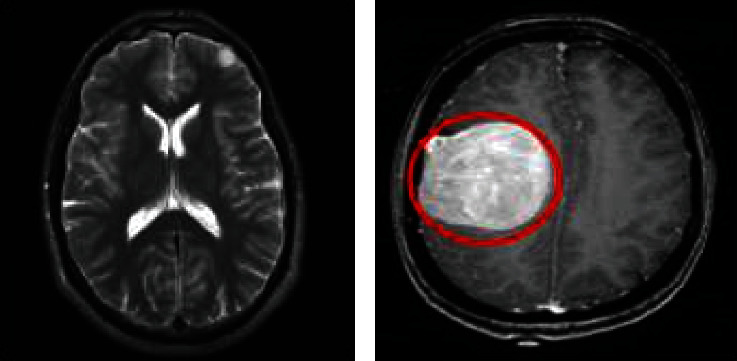
Visual insights of brain MRI images: (a) normal brain and (b) tumorous brain.

The brain is one of the most significant and complex body organs, containing billions of cells. Brain tumors cause deoxyribonucleic acid (DNA) damage in brain cells, which can lead to brain cancer. A brain tumor can also develop other cancer-related symptoms in the body. The brain tumor is influenced by the uncontrolled separation and proliferation of irregular cell types inside or surrounding the brain. This cell group affects the operation of the brain and regular cells, resulting in irreversible brain damage and even mortality [1]. The magnetic resonance imaging (MRI) pictures of a healthy and tumorous brain are shown in Figures 1(a) and 1(b), respectively.

In 2019, nearly 86,000 fresh cases were discovered, and currently, almost 700,000 people are globally suffering from brain tumors. Since 2019, nearly 16,830 people have died as a result of brain tumors, with a life expectancy of 35% [2]. According to CancerNet [3], brain and other nervous system cancers are the tenth leading factor in mortality in humans. There are more than 120 malignancies in the brain and central nervous system (CNS) [4]. Specialists prefer MRI because it can capture complete organ images, tissues, joints, and the entire inner body structure using intense magnetic fields and radio frequency signals. Some tumors are durable and unlikely to return due to the involved cells and impacted portion of the brain, whereas others may be dangerous, spread quickly, and be more difficult to treat. Benign (lower levels I and II) and malignant (higher levels III and IV) brain tumors can be found [5]. Accurate prediction of brain tumor types is critical in a treatment setting. Brain tumor identification and segmentation in MRI are critical areas of research in image analysis. Brain tumor's size, density, shape, position, and growth rate vary considerably [6]. Furthermore, different pathological types of tumors may appear similar [7]. When diagnosing brain tumors from MRI images, doctors typically use traditional assessment, which often suffers from human subjectivity. Hence, an automated computer-aided strategy proved to be effective in early-stage brain tumor identification with high precision.

The goals of this research are to investigate the importance of various image processing techniques on MRI images for determining brain tumors. Hence, an automated contour-based transfer learning approach using a VGG-16 pretrained model is presented here. The transfer learning model offers more effectiveness at enhancing generalizability, decreasing learning times, and boosting performance on less labeled data [8]. The fixed architecture of the VGG-16 model could often cause overfitting on smaller datasets, particularly medical data; hence, three dropout layers are introduced after each of the dense layers. This aids in tackling overfitting, capturing high-level features and relationships within the data by randomly dropping out neurons during the training phase. A thresholding-based segmentation method is also introduced following contour cropping. The segmentation technique assisted in retaining the necessary information to efficiently detect and classify brain tumors from MRI images. Finally, the model was validated on unseen data to determine its generalizability, robustness, and real-world applicability. This test case strengthens this study's significance in real-world scenarios. As a result of this study, medical professionals will be better equipped to diagnose and treat patients by employing MRI images and a deep learning technique to identify brain cancers. Overall, the noteworthy contributions of this study include the following:

- This study tackled the data diversity and robustness of the deep learning model by meticulously utilizing MRI image processing techniques. Particularly, the contour identification and cropping with the maximum area and a thresholding-based tumor segmentation assisted in retaining the maximum notable features from images
- This study introduces a new thresholding-based MRI segmentation procedure to segment brain tumor from the contour-cropped image
- The key innovation lies in enabling three trainable convolutional layers and incorporating three dropout layers within the VGG-16 transfer learning model to offer an advancement in the ContourTL-Net model for detecting and classifying brain tumors. The incorporation of the dropout layers within the model mitigates overfitting and improves generalization for efficient handling of unseen MRI data with high efficacy

The rest of the study is arranged as follows: the literature review is stated in Section 2, and the suggested methodology is categorized into subsections and briefly discussed in Section 3. Brain contour detection is discussed in Section 3.1.3. The proposed VGG-16 transfer learning model for effective brain tumor detection is discussed in Section 3.2.2.  Section 4 contains four subsections, including the dataset in Section 4.1 for data acquisition and descriptions. The experimental analysis is discussed in Section 4.4. The results are discussed in Section 5 concerning different methods and the datasets. The future scopes and limitations are delineated in Section 6. Finally, the conclusion is discussed in Section 7.

## 2. Literature Review

Preprocessing, segmentation, and feature obtaining are some of the image preparation techniques used to detect and categorize brain tumors. Machine learning (ML) algorithms are widely utilized for detection and classification, relying on features identified by feature obtaining methods. To process a large amount of data, ML algorithms require more computational power and time. Advanced deep learning models based on transfer learning achieved higher classification performance when employed to categorize MRI images without operating separate feature extraction strategies [4, 9]. Several image processing operations are performed before feeding the images into a deep learning model. The preprocessing stage reduces artifacts that could mislead the model and also increases its efficiency. To reduce noise, the authors of [10, 11] proposed boosted nonlocal mean filters and anisotropic diffusion filters, respectively. A similar outcome was achieved using the level set method for bias field correction with a median filter proposed in [12]. For segmentation, researchers used various algorithms, for example, histogram thresholding-based segmentation [13], Berkeley wavelet transformation- (BWT-) based tumor segmentation [14], morphological operation-based segmentation [4], custom mask region-based segmentation [12], and a multilevel segmentation combining optimal thresholding and watershed segmentation technique [15]. A color-based separation procedure is employed by [16] to segment FAIR and T1C-type MRI images. In most cases, the extracted features from the segmented sample image were fed into a convolutional neural network model (CNN) for categorization and achieved adequate accuracy. Furthermore, the authors in [17] proposed an optimal Kapur's thresholding-depending segmentation with a deep neural network (OS-DNN) model to effectively detect the affected region. Their proposed model had a maximum sensitivity of 97.94%, a specificity of 98.08%, and an accuracy of 98.02%. The ANN [13], support vector machine (SVM) [11, 16, 18, 19], and k-nearest neighbor (KNN) and random forest (RF) [20] are popularly used ML classifiers for brain tumor categorization. A chi-square test for feature selection with gray-level cooccurrence matrix (GLCM) texture characteristics allowed the SVM to perform better in [19]. A lightweight ensemble model in [21] incorporates numerous XGBoost decision trees to detect brain cancer from MRI images. The authors extracted intensity, texture, and shape features from the images to classify four grades of patients and obtained 93% accuracy. However, they lack in assessing their model on diverse datasets to strengthen the practical implementation of the work. Transfer learning was also used in the [18] brain tumor classification model to extract discriminative visual features and patterns. The experimental result with the fine-tuned VGG-16 shows an accuracy of 98.69%. The authors of [22] presented a comprehensive performance analysis of transfer learning-based pretrained models such as VGG-16, ResNet-50, and Inception-v3 models for automatic prediction of brain tumors. Using the VGG-16 transfer learning model, they achieved the highest accuracy of 96%. The authors [5] train and test deep transfer learning methods VGG-16, VGG-19, ResNet-50, and densenet21. Using the “adadelta” optimization algorithm, they discovered the highest performance accuracy of 99.02% with the ResNet-50 model.

VGG-16 efficiently recognizes patterns with high precision and robustness; however, its performance is sensitive to the collection size and hyperparameter choices [23]. In order to attain optimal performance, it is therefore difficult to fine-tune the model in addition to choosing the dataset and adjusting the hyperparameters. Authors in [24] proposed a CNN-based computerized brain tumor detection system where geometrical and statistical data augmentation strategies are used on brain tumor MRI images to improve CNN performance. They showed a comprehensive analysis of their model on different datasets, where they analyzed the model on 72% of unseen data and achieved 98.81% average accuracy. For the pathological brain image classification, the authors [25] proposed a fully automated process to investigate the potential of several pretrained DCNN architectures with the transfer learning approach. Various pretrained DCNNs, such as AlexNet, VGG-19, ResNet-50, GoogLeNet, Inception-ResNet-v2, VGG-16, ResNet101, Inception-v3, and Inception-ResNet-v2, were used. A hybrid network combination of U-Net and VGG-16 with transfer learning is proposed by authors [26] to simplify the U-Net architecture. To classify the brain tumor images, different traditional and hybrid ML models were built and analyzed [27]. In the experimental analysis of the 16 transfer learning models, they finally proposed a stacked classifier, VGG-SCNet (VGG stacked classifier network). In the data analysis, the complete back portion of the images is removed using contour identification, and each image is cropped using contour detection. The transfer learning model is a predefined procedure for the deep learning model to speed up the training time. Besides, deep learning methods are very useful in object detection and classification [4, 28].

The authors [29] presented an automated ultralight brain tumor detection (UL-BTD) technique on top of a novel ultralight deep learning architecture (UL-DLA) for deep features. Then, for multiclass categorization of tumors, they employed an SVM which obtained an average detection rate of 99.23%. However, an expert opinion on general trial was needed for clinical evaluation. They also mentioned that an explainable artificial intelligence technique could be analyzed to discover complex prediction and decision-making strategies. The study [30] addressed two issues: (i) clinical brain tumor segmentation from homogenous data with high efficiency and (ii) heterogonous data analysis by constructing a multiscale dilated feature upsampling network (MDFU-Net). Incorporating multiscale detailed features (MDF) in the encoder module significantly enhanced segmentation performance. Then, a decoder module was designed to process the dense spatial MDF. Though this model showed considerable performance, however, for the heterogonous data, it showed a decrease in sensitivity. An advanced deep learning model, YOLOv7, has been operated by [31] to perform transfer learning on a large MRI collection to detect multitype tumor location precisely and achieve 99.5% accuracy. The convolutional block attention module (CBAM) was added to the YOLOv7 model to obtain these results. However, they did not discuss how the model performs on diverse characteristics of MRI images.

## 3. Method and Model

**Figure 2:**
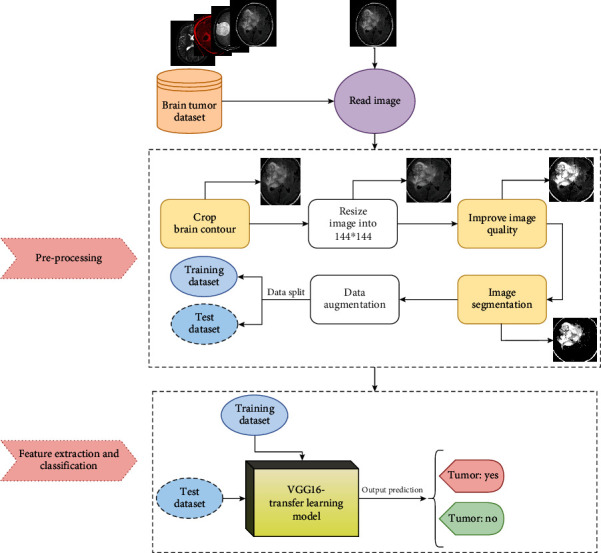
The detailed proposed architecture for efficient brain tumor detection. This begins with preprocessing the input MRI images and then employing transfer learning with a VGG-16 pretrained model for robust feature gathering and effective tumor identification.

The suggested research work is separated into two stages: the first is preprocessing, and the second is feature extraction and classification. Brain contour cropping is included in the preprocessing step. Following image enhancement, the thresholding technique is used for segmentation. The preprocessing phase aids in the reduction of artifacts that could cause the transfer learning model to be misled. In the second phase, a transfer learning model is created. To accomplish this, we used a pretrained model that outperforms other models for brain tumor detection and classification [18, 22, 26]. The pretrained model's initial weights are obtained from the popular “ImageNet” dataset so that the model can be learned effectively with fewer time and epochs. To evaluate the proposed transfer learning model, two datasets were used. The proposed procedure is illustrated in the schematic diagram in Figure 2.

### 3.1. Preprocessing

The primary goal of medical image processing is to clean MRI images and reduce artifacts to obtain a better feature of the represented image by using a variety of image processing techniques such as brightness correction, contrast enhancement, noise reduction, morphological operation, and unnecessary object removal. To complete this research, a region-based segmentation technique is utilized due to its computational efficiency and simplicity nature [32]. The segmentation strategy has been enhanced by extracting the contour area from the image. This shaped our suggested model to outperform the existing methods.

#### 3.1.1. Grayscale Conversion

A grayscale image is more convenient than an RGB image to process as it has only one channel. To ensure ease of computation, this study initially modified the RGB image to grayscale to perform more complex operations in less time for various image processing tasks such as morphological operation and segmentation. Then, the Gaussian filter was used on the image to enhance the object's boundary to further reduce the sudden color transformations that are normally separated and not necessary to understand the image [33].

#### 3.1.2. Binarize Image

Thresholding is a technique for binarizing an image that concentrates on objects or locations of distinct interest in an image. In this method, a threshold value *T* is chosen, and all pixel values less than *T* are set to 0, otherwise to 255. In order to extract image features, morphological operations support the legation and recital of region shapes such as borders, skeletons, and convex hulls. The morphological operation requires a structuring element. In practice, the structuring characteristic is usually much lesser than the image and is only rarely used as a 3 × 3 matrix. The two most important morphological operations are erosion and dilation. (a) Erosion reduces the size of foreground objects and increases the size of foreground holes by removing pixels on object boundaries. (b) Dilation works by object expansion by adding pixels to the boundaries of objects in a picture.

#### 3.1.3. Contour Detection

**Figure 3:**
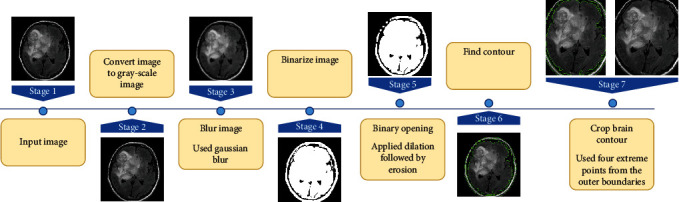
Process of brain contour cropping, showcasing image preprocessing techniques and enhancements to effective contour detection and cropping.

A contour is defined as a simple curve that connects all straight points (along the boundary) that have the same color or intensity. Tumor segmentation might be affected due to the sensitivity to the selection of seed point preference [32]. Initializing seed points in the form of contour guides the effective segmentation process [34]. This has significantly improved model performance as well as accuracy [35]. We obtain a median filtered image suitable for contour detection after preprocessing the images depicted in the earlier section. The large white portion within the black area is seen by analyzing the image obtained in the previous step. This is used frequently as one of the several techniques for locating blobs (large binary objects) in a picture using OpenCV [36]. In this work, CV_RETR_EXTERNAL mode and CV_CHAIN_APPROX_SIMPLE method are utilized. CV_RETR_EXTERNAL mode retrieves only the extreme outer contours, and CV_CHAIN_APPROX_SIMPLE compresses horizontal, vertical, and diagonal segments and leaves only their endpoints. After identifying the connected contours of the image, the outer boundaries of the connected contour are calculated. Then, cropping was done by selecting the most significant contour as shown in Figure 3. This enhanced the performance and precision of object identification significantly in our work.

#### 3.1.4. Enhancement

**Figure 4:**
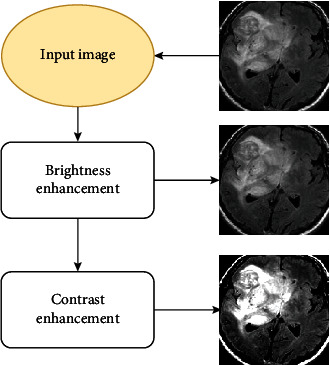
Illustration of the brain MRI image quality enhancement process.

After detecting and cropping contours in the MRI images from the last section, an image resizing operation is performed to reduce the input images to 144 × 144 size. Then, image enhancement strategies are employed to eliminate anomalies between image areas and improve visual clarity. Effective image enhancement reduces redundant pixels and noise, enhancing separation between bright and dark regions [37]. To utilize these benefits, the resized images are subjected to brightness and contrast enhancement techniques, which increase the difference between abnormal and normal cells (Figure 4).

#### 3.1.5. Segmentation

Finally, a region-focused segmentation strategy is performed to divide the images into subparts which is used to extract the tumor. In this phase, the threshold segmentation is operated to compare each pixel value in the MRI image to the threshold value. If the pixel value is less than the threshold value, it becomes zero; if it is greater than or equal to the pixel value, it remains unchanged. The segmentation procedure is described by equation (1). By comparing a threshold value, this procedure removes pixels other than the tumor area.

(1) $$\begin{array}{c} f\left(x,y\right)=\left\{\begin{array}{cc} f\left(x,y\right), & \text{if }f\left(x,y\right)\geq \text{threshold},\\ 0, & \text{otherwise}. \end{array}\right. \end{array}$$

**Figure 5:**
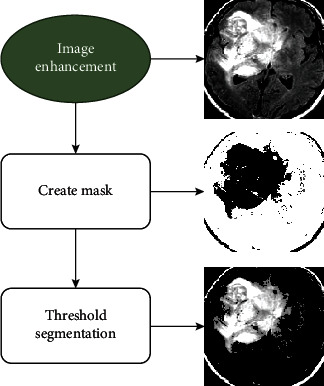
Tumor segmentation process in brain MRI images through advanced thresholding techniques assisting the model to gather significant features from the images.

Except for the spatial attributes of an image, thresholding is an efficient technique for image segmentation [38]. It minimizes the overall computational complexity and works faster than other methods. The tumor segmentation process is illustrated in Figure 5, where the threshold value is computed using Algorithm 1.

### 3.2. Feature Extraction and Classification

The CNN has an exceptional feature extraction technique in terms of speed and efficacy when compared to the existing conventional feature obtaining strategies. CNN extracts input image features, which are then utilized by another neural network to classify the images. In this study, the VGG-16 deep neural architecture is used for feature extraction and classification.

#### 3.2.1. Transfer Learning

**Figure 6:**
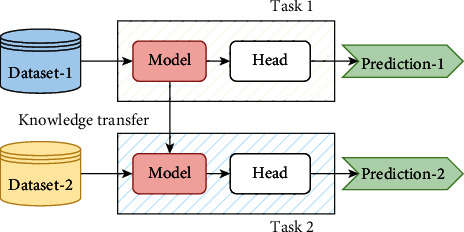
Visual explanation of the transfer learning procedure.

Transfer learning solves problems by applying previously learned knowledge to a new task. Rather than creating a model from scratch, the deep transfer learning model utilized the learned knowledge from a massive amount of data such as “ImageNet” weights for image classification, to transfer information from the source domain to the target domain [26]. The transfer learning process is depicted in Figure 6. This has a positive impact on many difficult domains to improve due to a lack of training data [39]. Transfer learning has the advantage of reducing overall training time because it uses the weights of a pretrained model [5]. Using the weights of an earlier learned model with a large amount of data yields more consistent and high-performance results [5]. Furthermore, no feature extraction step is required [25].

#### 3.2.2. VGG-16 Transfer Learning Model

**Figure 7:**
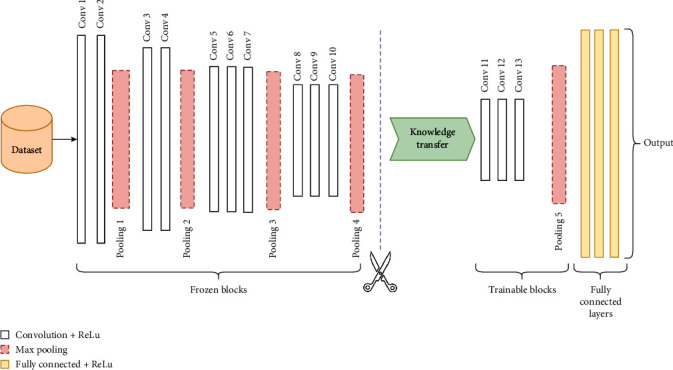
The proposed VGG-16 transfer learning model, designed by freezing ten initial convolutional layers and fine-tuning the three subsequent convolutional layers with the investigated dataset, enhances precision in brain tumor classification.

Along with other pretrained models DenseNet121, ResNet50, and Inceptionv3, the VGG-16 model is a widely utilized pretrained DCNN model for analyzing medical images and automatic feature extraction and classification [40–42]. Compared to other models, the VGG16's simplicity and uniformity in architecture make it a good fit for transfer learning applications and convenient training complexity [43]; particularly, it carries excellent generalization capability in the medical field where domain knowledge is essential. In this experiment, a transfer learning network is designed using the well-known VGG-16 pretrained CNN model. The model, as proposed in study [44], consists of total 16 layers, with 13 layers performing convolution (Convo) and the remaining three layers being fully connected. It contains 138 million trainable parameters and achieves 92.7 percent top 5 test accuracy in the “ImageNet” dataset. The VGG-16 model transfers the extracted features to the fully connected layer to classify the image after extracting features from the image (Figure 7).

To transfer knowledge from previously trained layers with the “ImageNet” dataset, the first ten of its pretrained Convo layers were kept frozen, preventing them from being trained during the training phase. The remaining three layers were prepared with the provided image data. There were 11,602,818 trainable parameters in total. It accepts dimension input of 14 × 144 × 3. Stride 1 is used with 3 × 3 filters in the Convo layers. With the 2 × 2 window size and stride 2, five max-pooling operations are performed. A total of three dropout layers are used after each dense layer during training to set the input units to 0 at random with a frequency of rate. It aids in the prevention of overfitting [45]. In the final layer, a softmax activation function is employed to specify which class the network outputs belong to. The softmax activation function yields a probability value ranging from 0 to 1 (Equation (2)). Rectified linear unit (ReLU) activation function (Equation (3)) is used in all hidden layers. The architecture related to VGG-16 transfer learning is depicted in Figure 7.

(2) $$\begin{array}{c} \text{Softmax}\left(z_{i}\right)=\frac{e^{z_{i}}}{\sum_{j=1}^{k}e^{z_{i}}}. \end{array}$$

Here, *z* represents the output layer's value from the neuron and

(3) $$\begin{array}{c} F\left(x\right)=\max\left(0,x\right). \end{array}$$

## 4. Performance Evaluation

Python was used to develop and test the proposed methodology's experimental justifications. To code, test, and analyze model performance, several libraries, particularly TensorFlow and Keras, were embedded in the system. The proposed VGG-16 architecture is trained and tested using Colab Notebook on the Google Cloud Platform. This platform includes a 12 GB NVIDIA Tesla K80 GPU that can run in the background for up to 12 hours and is tightly integrated with Google Drive.

### 4.1. Dataset

A set of experiments has been performed on publicly available two distinct datasets: dataset-1 (BR253) [46] and dataset-2 (BR35H) [47]. These datasets are collected from the “Kaggle Website” and contain diverse patient MRI image data of different dimensions. Both the datasets are labeled into two classes (yes and no) depending on the tumor presence in the image.

**Figure 8:**
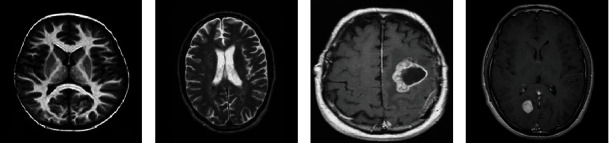
BR253 MRI image: (a, b) nontumorous brain and (c, d) tumorous brain.

**Figure 9:**
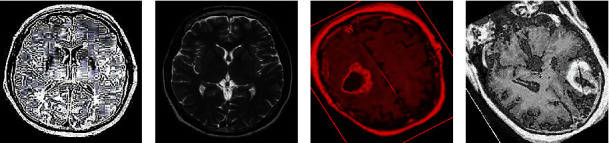
BR35H MRI image: (a, b) nontumorous brain and (c, d) tumorous brain.

The BR253 collection contains 253 brain MRI images, out of which 98 are normal, while the remaining 155 are abnormal. Though most image formats are JPG, however, a few PNG images are also seen in this dataset. The BR35H dataset contains a total of 3000 brain MRI images where 50% are denoted as normal and the rest 50% are labeled as abnormal MRIs. All the images seen in this dataset are in JPG format. The BR35H dataset also includes T1-weighted and T2-weighted image sequences. The BR35H dataset is rated as 7.5 in terms of usability. The data usability rating is based on tagging, data overview, licensing, description, ease of maintainability assurance, machine-readable file formats, metadata, and public kernel availability. Several images from the BR253 and BR35H datasets are consecutively shown in Figures 8 and 9.

### 4.2. Data Augmentation

**Figure 10:**
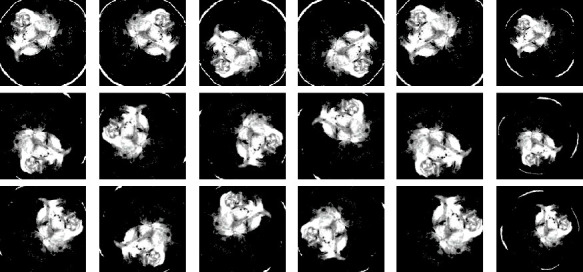
Brain tumor data augmentation sample image: (a) rotation, (b) flip vertical, (c) flip horizontal, (d) flip horizontal-vertical, (e) zoom in, and (f) zoom out.

This study employs a data augmentation procedure to expand the dataset size. This ensures the deep learning model's robustness, allows a comprehensive assessment, and extends its generalizability to previously undiscovered scenarios [48]. Data augmentation involves increasing the amount of data in a set through various transformations. Here, different image variations are generated through the data augmentation techniques to reduce model overfitting during the training period and increase detection accuracy for unseen data. The basic techniques for increasing the amount of data are rotation, flipping, and zooming (i.e., zoom in, zoom out). The rotation is carried out using Equations (4) and (5). The image is also mirrored from the vertical and horizontal directions, as described in Equations (6) and (7).  Figure 10 illustrates examples of image augmentation applied to brain images.

(4) $$\begin{array}{c} R_{\theta }=\left[\begin{array}{cc} \cos\theta & -\sin\theta \\ \sin\theta & \cos\theta \end{array}\right], \end{array}$$

(5) $$\begin{array}{c} \text{B}{\text{T}}_{\text{rotate}}\left(X,Y\right)=\text{B}{\text{T}}_{R\theta }, \end{array}$$

(6) $$\begin{array}{c} \text{B}{\text{T}}_{\text{flip}-\text{v}}\left(X,Y\right)=\text{BT}\left(X,-Y\right), \end{array}$$

(7) $$\begin{array}{c} \text{B}{\text{T}}_{\text{flip}-\text{h}}\left(X,Y\right)=\text{BT}\left(-X,Y\right). \end{array}$$

### 4.3. Training and Testing

Evaluating a model's validation is crucial when analyzing augmented and nonaugmented data. Augmentation transforms data to expand the dataset; however, the model's robustness in real-world applications can be discovered by using unaltered original data, as the augmented medical image might introduce artifacts that are absent in the original collection. Validating on the nonaugmented dataset ensures that the model's predictions align with the original medical image's characteristics. This also uncovers the model's performance on the same dataset for two scenarios (i.e., augmented and nonaugmented). This suggested model has been evaluated on two datasets, BR253 and BR35H, in four ways. The system first used data augmentation techniques to increase the image numbers in the BR253 and BR35H datasets; then, four distinct test scenarios, (i) BR253, (ii) BR35H, (iii) BR35H dataset with no augmentation (BR35H-NA), and (iv) unseen cases, were designed, as shown in Table 1. The first three datasets BR253, BR35H, and BR35H-NA utilized 80% of the total images for training, 20% of the training images for validation, and the final 20% of total images for testing the model. For the final model evaluation scenario (i.e., unseen case), both the BR35H and BR253 datasets are utilized. In this case, the BR35H and BR253 collections are augmented to generate a total of 24000 and 2024 images, respectively. For training and validation, the augmented BR35H dataset was divided into two halves for training and validation: 70% of the overall dataset was used for training, and 30% was used to validate the model. Finally, for the unseen scenario, the model has been tested with completely unknown 2024 images from the augmented BR253 dataset.

### 4.4. Experimental Analysis

In this study, two types of images were classified using the VGG-16 transfer learning architecture: brain tumor and nonbrain tumor. This fine-tuned model extracts relevant features from images using learned weights from the “ImageNet” dataset and the “RMSprop” optimizer with a learning rate of “2*e*-5”. The “sparse categorical cross-entropy” loss function is used to compute the quantity that the VGG-16 model should seek to minimize during the training phase. This loss function labeled the output as an integer (0, 1, 2, 3… and so on). For this study, all the hyperparameters of the model were chosen based on the sensitivity of the dataset during training the model. The model extracted features from the MRI images, which were then classified using a three-layer fully connected neural network. To ensure the robustness of our findings, multiple evaluations of our deep learning model have been conducted on the same dataset with random train-test-validation splits. This operation was repeated three times to observe the variability in outcomes and assess the consistency of the model's performance. By employing this approach, this study is aimed at offering a more comprehensive understanding of the model's generalization capability and at mitigating the influence of specific random splits on the mentioned evaluation metrics. During the training phase, the dropout layers were used to randomly drop some connections in order to provide more generalizability. Early stopping with dropout aids in addressing model overfitting [45]. Overfitting appears when the model trains too well on the training sample, such that the model performs best on the training sample but not well on the test or unknown sample [45]. At this point, the validation loss begins to decrease before increasing again. Seven performance assessment metrics, including (i) sensitivity or recall, (ii) specificity or true negative (TN), (iii) precision or positive predictive value (PPV), (iv) NPV, (v) accuracy, (vi) *F*1-score, and (vii) ROC-AUC, were employed to evaluate the suggested methodology. ROC-AUC is an acronym that stands for “region of the curve” and “area under the curve.” Sensitivity and specificity both assess the model's ability to correctly classify positive and negative cases, respectively. Sensitivity pertains to the model's performance in identifying true positive cases, such as images with brain tumors, while specificity refers to its performance in identifying true negative cases, for instance, images without brain tumors or normal cases. Precision is calculated by dividing the number of true positive predictions by the sum of true positive and false positive predictions, representing the accuracy of positive predictions within the positive class. The *F*1-score is a harmonic mean of precision and recall, providing a balanced measure of a model's performance across both precision and recall. It serves as a comprehensive assessment indicator for the general performance of the model [48]. Accuracy, on the other hand, measures the ratio of correctly predicted instances to the total number of instances, providing an overall assessment of the model's correctness. The metric ROC-AUC tests a model's capabilities by visually discriminating between model classes at various threshold points. The AUC is a measure or degree of separability. AUC is the volume of the area of the unit square; it is always between 0 and 1. The greater the AUC, the more accurate the model. AUC value should be close to 1 for better classification performance [49]. The best ROC-AUC value is considered in this investigation to determine the model's fairness.

As the suggested system is based on contour detection, after successful contour spotting and segmentation in the preprocessing step, the images are fed into a VGG-16-based transfer learning model. The tumor detection performances of the model were examined on the BR253, BR35H, and BR35H-NA datasets, and then, the clinical significance was investigated by assessing the model on the unseen data. The dataset BR35H is used to train the model for the unseen scenario, and the dataset BR253 is used to test the unseen data. The model was run three times on each dataset individually, and the results are shown in Tables 2–4. The ROC-AUC and validation loss curves guided the study to determine the optimal outcome among all.

The result comparison for the suggested model with the existing models for dataset BR253 is shown in Table 5. Although the enhanced model in [50] has higher specificity (0.67%) compared to other models, our suggested ContourTL-Net model surpasses the existing model's sensitivity and accuracy by 4.93% and 2.5%, respectively, which indicates its ability to better identification of true positive cases for the specific BR253 dataset. While comparing accuracy and sensitivity across Table 5, our proposed model achieves the highest accuracy (99.51%) and sensitivity (99.63%), outperforming the second-best accuracy (98.02%) by the OS-DNN model [17] by 1.49% and the second-best sensitivity (99.1%) by the VGG-SCNet model [27] by 0.53%. This strengthens the suggested model's capability to effectively identify positive and negative brain tumor cases and enhances the detection accuracy particularly BR253 data.

Table 6 compares the performance of the proposed methodology with augmentation and without augmentation with state-of-the-art methods for the BR35H dataset. The proposed model achieves superior results compared to the no-augmentation model across all metrics. These high values indicate the model's ability to accurately identify brain tumors while minimizing false positives and negatives. Even though there has not been much work done on the BR35H dataset to compare positive and negative case identification performance, the results in Table 6 demonstrate the accuracy comparison with the existing works. The suggested model with augmentation achieved 99.94% accuracy, surpassing the closest result by DCNN with SGD optimization model [51] by 0.94%.

For the clinical study, an unseen dataset BR253 with augmentation (2024 images) is evaluated by the proposed ContourTL-Net model, which was trained with BR35H with augmentation (16800 images) and obtained 99.46% accuracy. We took five sets of testing results for the unseen data, and the results of testing the unseen data are shown in Table 7. To compare with the state-of-the-art techniques, the best unseen case outcomes are adapted from Tables 7 and 8. This model's accuracy beats the existing study, CNN model [24] and isolated CNN classifier [52], by 3.21% and 2.26%, respectively, as illustrated in Table 8. The suggested model showcases its extensive ability to correctly identify all positive cases of brain tumors in the unseen dataset by achieving 100% sensitivity and surpasses the CNN model [27] by 5.2%. The model's precision of 99.12% surpasses the existing CNN model [27] by 0.79% indicating a high reliability with a low rate of false positives, enhancing its efficacy in brain tumor identification.

## 5. Discussion

**Figure 11:**
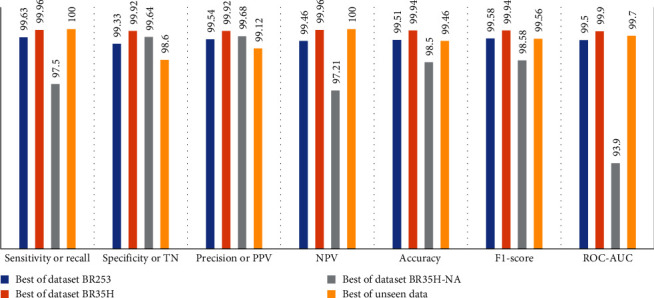
Proposed model result comparison for variety of data.

From Table 2, S/L no. 2 is identified as the best result among all three test results for dataset BR253.  Table 9 shows that the validation loss curve for test 1 has a sudden considerable change at epoch 20 and the validation loss curve for test 3 has a sudden significant change at epoch 11. In contrast, test 2 does not have a too big sudden change in the epoch. From Table 3, S/L number 3 is identified as the best result among all three results for dataset BR35H.  Table 10 shows that the validation loss curve for test 1 has three sudden big changes at epochs 4, 21, and 30, while the validation loss curve for test 2 has four sudden huge changes at epochs 14, 22, 24, and 29. Although test 3 contains three validation loss changes, it still attempts to decline at 30 epochs. In Table 4, S/L number 1 is identified as the best outcome for dataset BR35H with no augmentation when compared to the other two validation loss curve S/L nos. 2 and 3 from Table 11. The performance of the model on a range of data is illustrated in Figure 11. The suggested model is evaluated on seven metrics, and the BR35H dataset results show a significant dominance compared to the other datasets for specificity, precision, accuracy, *F*1-score, and ROC-AUC, whereas the unseen case outperforms all other datasets for sensitivity (100%) and NPV (100%) metrics. In Table 12, test 4 has a superior validation loss curve than the other results. The validation loss for test 4 approaches the training loss.

The overall performance on the BR253 shows degradation compared to the BR35H dataset; this is due to having less data in the BR253 collection than the BR35H dataset. The BR35H with no-augmentation dataset's results also advocates this statement as it was unable to exceed any other outcomes with the original 3000 MRI images in its collection. As this study's objective was to detect and classify the unseen data case with high efficiency thus enhancing the robustness and data diversity, tackling the model's evaluation of the unseen case states the strengths of this study by achieving considerable performance on the evaluation metrics sensitivity (100%), specificity (98.6%), PPV (99.12%), NPV (100%), accuracy (99.46%), *F*1 (99.56%), and ROC-AUC (99.70%). This was possible by introducing a modified new thresholding-based MRI segmentation and fine-tuned VGG-16 transfer learning model. However, before going for clinical validation, this model further needs to be tested with real-life clinical data and increased characteristics of brain MRI images.

## 6. Future Works and Limitations

This study presents a ContourTL-Net, by incorporating image preprocessing and utilizing a pretrained deep learning model VGG-16. During the preprocessing phase, this study employs contour cropping followed by segmentation to feed the pretrained network. The model utilizes pretrained “ImageNet” weights to facilitate the transfer learning process. While the overall performance of the model on the unseen dataset is satisfactory, further research into its prediction and decision-making mechanisms is necessary, especially with a more extensive dataset. Due to hardware limitations, our investigation was confined to the [46, 47] datasets. This emphasizes the need for additional data to facilitate clinical implementation. Incorporating tumor substructure segmentation with larger data such as BraTS2020 [55] could be explored in the future, to enhance patient survival prediction [56]. A review by an expert in the area of deep learning and medical imaging might be feasible to assess the proposed ContourTL-Net model's real-life applicability. Other recently innovated pretrained models such as YOLOv7 and EfficientNet can be explored to find a better transfer learning approach for tumor detection. This study implemented the transfer learning methodology using the VGG-16 architecture. However, no additional convolutional or attention mechanisms were incorporated that require extensively conducting the time and computational complexity. This additional layer inclusion might also be introduced and analyzed in future investigations.

## 7. Conclusion

In recent years, MRI images have been increasingly used for the efficient identification of brain tumors due to the growing risk of mortality associated with this condition. Early diagnosis of brain tumors can significantly reduce this risk; however, current diagnostic methods are time-consuming and rely on operator expertise; hence, to address these issues, recent studies include complex deep learning models that are lacking in experimentally validating on unseen MRI images. This study proposed a ContourTL-Net methodology for detecting and classifying brain tumors operating contour detection and a VGG-16 transfer learning model. The primary objective was to distinguish between healthy and abnormal brain tissues at an early stage by offering a deep learning model that tackles data diversity and ensures robustness. The model was evaluated on four distinct sources, with the best results chosen based on validation loss and ROC-AUC analysis. Additional evaluation criteria were used to compare the model's performance with existing methodologies. The model achieved high accuracy on the datasets, ranging from 98.50% to 99.94%. In addition, the model exceeded existing methods for detecting brain tumors on an unseen dataset, by 2.26% accuracy. For other cases, (i) in the BR253 dataset, the model outperformed the existing highest achieved accuracy by 1.49%, and (ii) in the BR35H dataset with augmented image, the model surpassed the existing top-scored model by 0.94%. Utilizing large contour cropping and thresholding segmentation assisted in retaining valuable features. This enhances our suggested model's generalizability, robustness, and overall performance when integrated with our fine-tuned VGG-16 transfer learning model featuring three dropout layers. Although the results vary across different datasets, the outcomes are consistent. Particularly, the unseen case evaluation of the proposed model validates its significance in detecting benign and malignant brain tumors in a clinical setup. Overall, the model's robust performance suggests its potential by offering a reliable tool for clinical brain tumor identification to revolutionize brain tumor diagnosis, enabling prompt intervention and improving patient outcomes. While the effectiveness of the proposed model is evident, however, before clinical deployment, validation by both clinicians and ML experts is recommended. Future research might utilize collaboration between medical and ML specialists to optimize the tumor segmentation process and integrate advanced deep learning techniques to investigate the proposed ContourTL-Net model's strength.

## Figures and Tables

**Algorithm 1:**
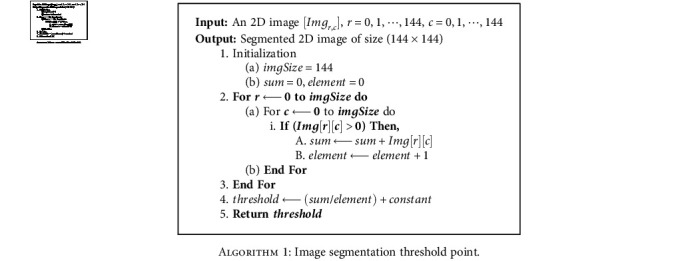
Image segmentation threshold point.

**Table 1:** Data partitioning: distribution and allocation of the four datasets for training, validation, and testing phases in the experimentation process.

S/L	Dataset	Total images	Trained images	Validation images	Target images
1	BR253	9108	5828	1458	1822
2	BR35H	24000	15360	4800	3840
3	BR35H-NA	3000	1920	480	600
4	Unseen case	26024 (augmented BR253 and BR35H)	16800	7200	2024

**Table 2:** Experimental result (%) analysis for BR253 dataset.

S/L	Sensitivity (recall)	Specificity (TN)	Precision (PPV)	NPV	Accuracy	*F*1-score	ROC-AUC
1	99.73	99.44	99.64	99.58	99.62	99.68	0.996
2	**99.63**	**99.33**	**99.54**	**99.46**	**99.51**	**99.58**	**0.995**
3	99.63	99.64	99.09	99.45	99.23	99.36	0.991

Significant performances are denoted in bold.

**Table 3:** Experimental result (%) analysis for BR35H dataset.

S/L	Sensitivity (recall)	Specificity (TN)	Precision (PPV)	NPV	Accuracy	*F*1-score	ROC-AUC
1	99.95	99.92	99.92	99.96	99.94	99.94	0.999
2	99.87	100	100	99.88	99.94	99.94	0.999
3	**99.96**	**99.92**	**99.92**	**99.96**	**99.94**	**99.94**	**0.999**

Significant performances are denoted in bold.

**Table 4:** Experimental result (%) analysis for BR35H dataset with no augmentation.

S/L	Sensitivity (recall)	Specificity (TN)	Precision (PPV)	NPV	Accuracy	*F*1-score	ROC-AUC
1	**98.44**	**98.57**	**98.75**	**98.22**	**98.50**	**98.59**	**0.939**
2	97.81	98.93	99.05	97.54	98.33	98.43	0.93
3	97.5	99.64	99.68	97.21	98.50	98.58	0.939

Significant performances are denoted in bold.

**Table 5:** Experimental result (%) evaluation of the proposed methodology with the state-of-the-art methods for BR253 dataset.

Methods	Sensitivity (recall)	Specificity (TN)	Accuracy
Improved model [50]	94.70	**100**	97.01
ResNet-50 model [53]	—	—	95.0
VGG-SCNet model [27]	99.10	—	—
UNet-VGG-16 model [26]	—	—	96.10
BrainMRNet model [39]	96.0	96.08	96.05
OS-DNN model [17]	97.94	98.08	98.02
**Proposed model**	**99.63**	99.33	**99.51**

Significant performances are denoted in bold.

**Table 6:** Experimental result (%) evaluation of the proposed methodology with the state-of-the-art methods for BR35H dataset.

Methods	Sensitivity (recall)	Specificity (TN)	Precision (PPV)	NPV	Accuracy
DenseNet-169-based FC layer model [54]	—	—	—	—	98.83
DCNN with SGD optimization [51]	—	—	—	—	99.0
Proposed model (no augmentation)	98.44	98.57	98.75	98.22	98.50
**Proposed model**	**99.96**	**99.92**	**99.92**	**99.96**	**99.94**

Significant performances are denoted in bold.

**Table 7:** Experimental result (%) analysis for unseen data.

S/L	Sensitivity (recall)	Specificity (TN)	Precision (PPV)	NPV	Accuracy	*F*1-score	ROC-AUC
1	100	99.49	99.68	100	99.80	99.84	0.997
2	99.44	99.62	99.78	99.11	99.51	99.60	0.995
3	99.76	98.47	99.04	99.61	99.26	99.40	0.991
4	**100**	**98.60**	**99.12**	**100**	**99.46**	**99.56**	**0.997**
5	99.92	99.36	99.60	99.87	99.70	99.76	0.996

Significant performances are denoted in bold.

**Table 8:** Experimental result (%) evaluation of the proposed methodology with the state-of-the-art methods for unseen dataset.

Method	No. of total test image	Sensitivity (recall)	PPV (precision)	Accuracy (%)
CNN model [24]	1265	94.80	98.33	96.25
Isolated CNN classifier [52]	—	—	—	97.20
**Proposed model**	**2024**	**100**	**99.12**	**99.46**

Significant performances are denoted in bold.

**Table 9:** Experimental result analysis for BR253 dataset.

Dataset	Loss curve	Accuracy curve	ROC-AUC	Confusion matrix
BR253 dataset, test 1	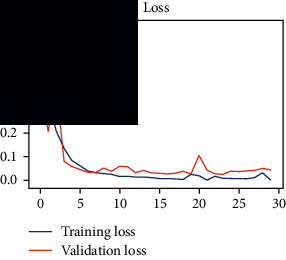	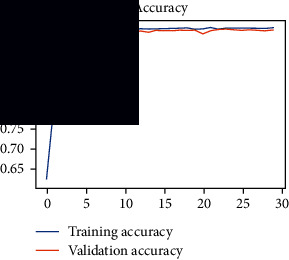	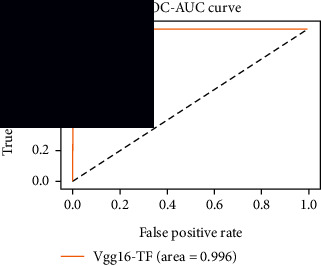	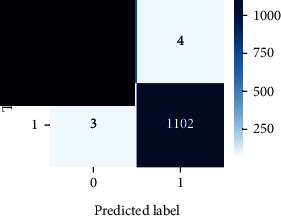
BR253 dataset, test 2	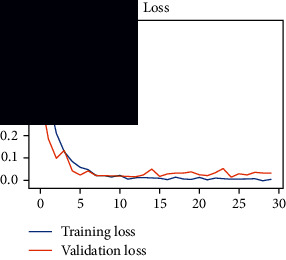	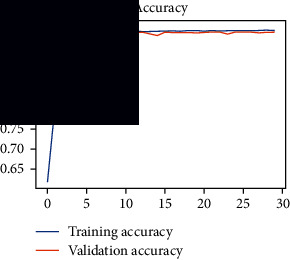	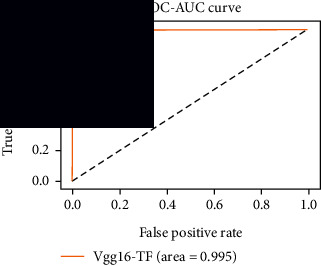	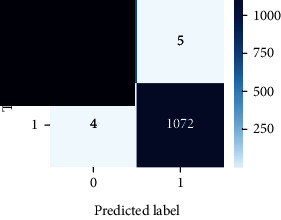
BR253 dataset, test 3	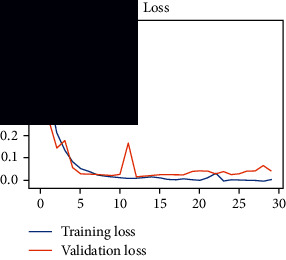	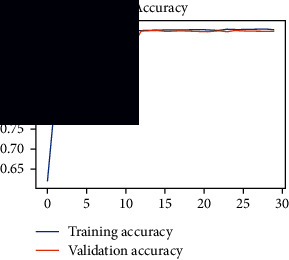	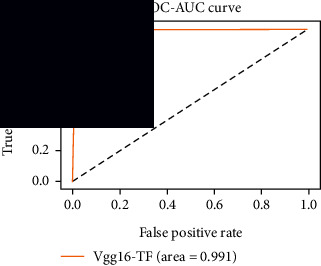	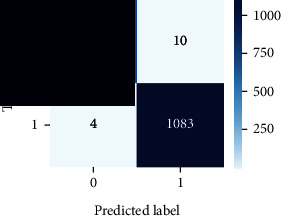

**Table 10:** Experimental result analysis for BR35H dataset with augmentation.

Dataset	Loss curve	Accuracy curve	ROC-AUC	Confusion matrix
BR35H dataset, test 1	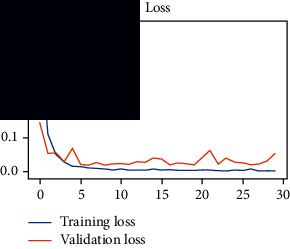	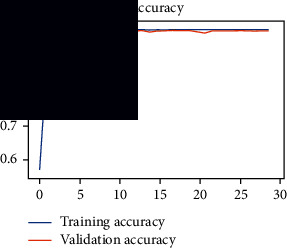	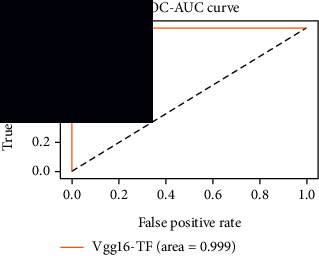	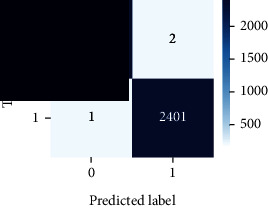
BR35H dataset, test 2	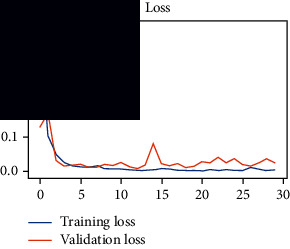	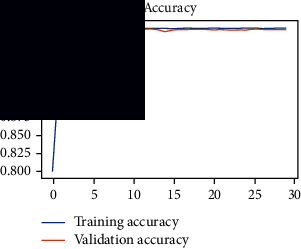	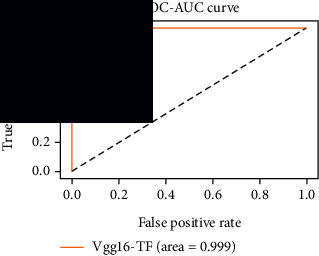	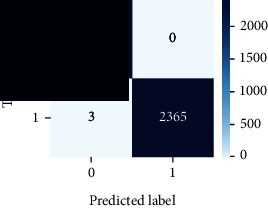
BR35H dataset, test 3	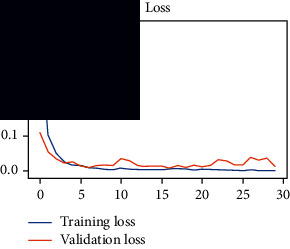	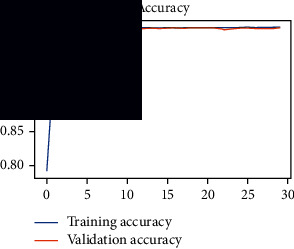	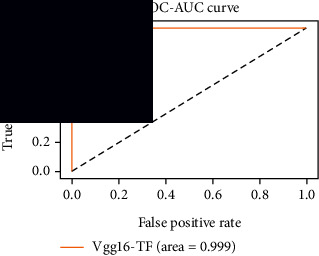	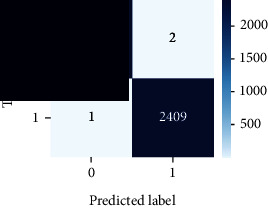

**Table 11:** Experimental result analysis for BR35H dataset with no augmentation.

Dataset	Loss curve	Accuracy curve	ROC-AUC	Confusion matrix
BR35H dataset, test 1	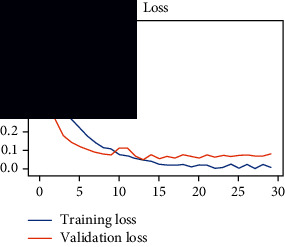	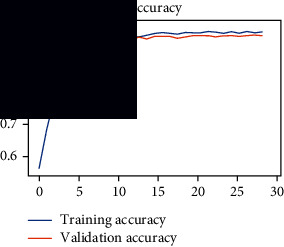	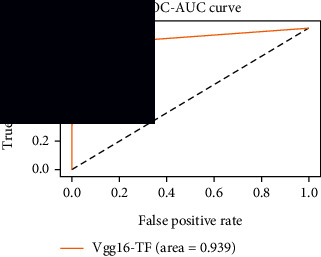	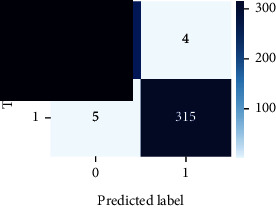
BR35H dataset, test 2	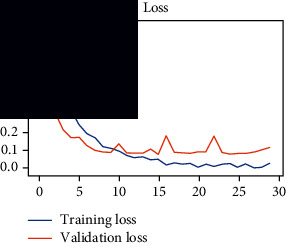	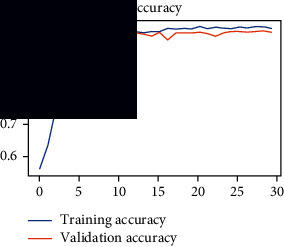	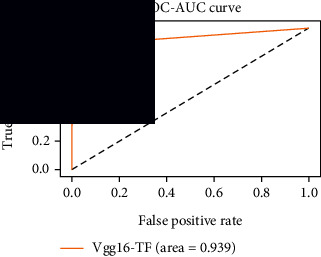	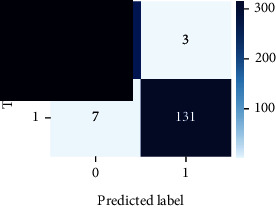
BR35H dataset, test 3	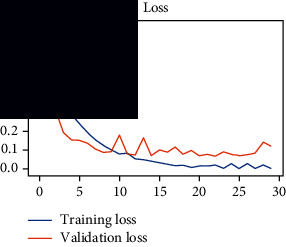	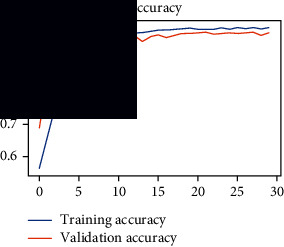	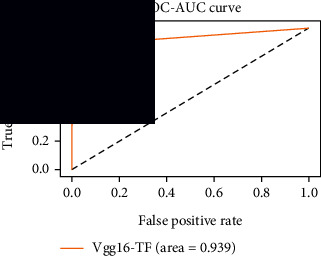	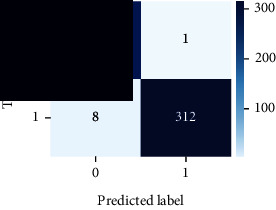

**Table 12:** Experimental result analysis for unseen data (i.e., train = BR35H and test = BR253).

Dataset	Loss curve	Accuracy curve	ROC-AUC	Confusion matrix
Unseen, test 1	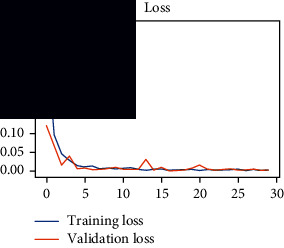	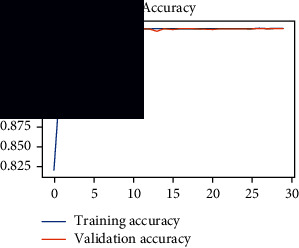	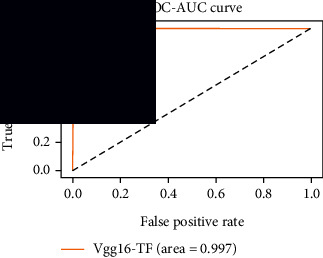	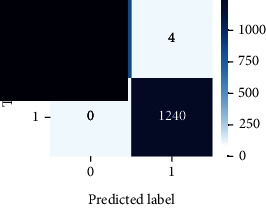
Unseen, test 2	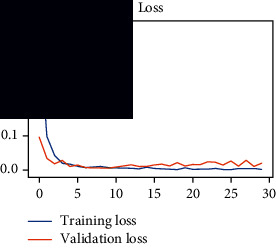	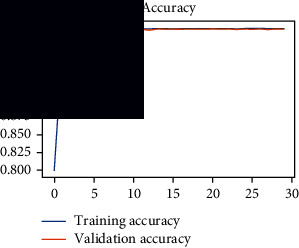	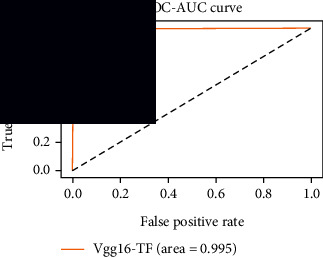	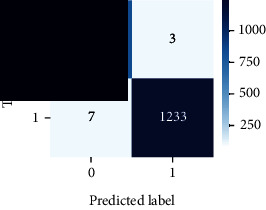
Unseen, test 3	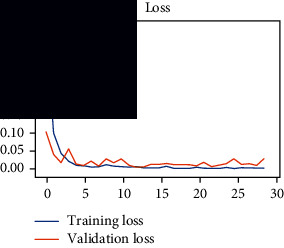	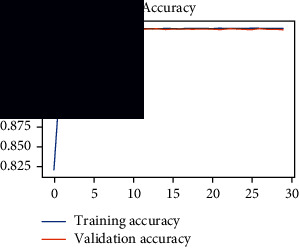	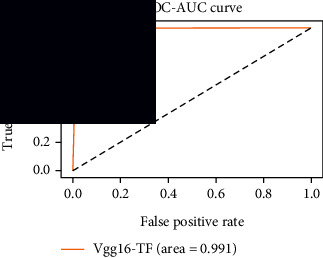	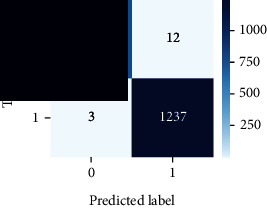
Unseen, test 4	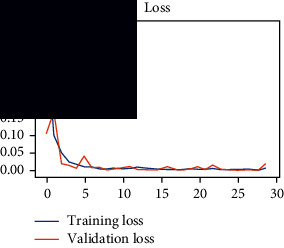	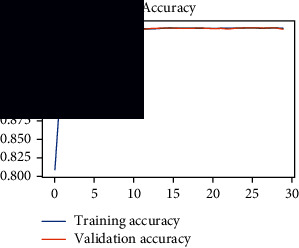	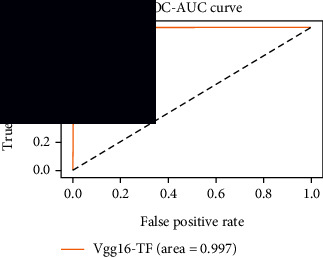	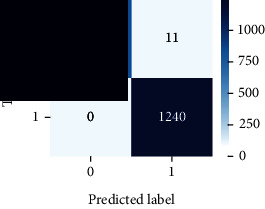
Unseen, test 5	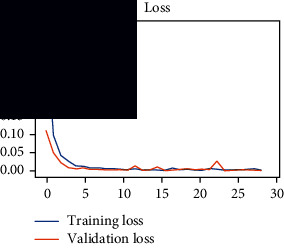	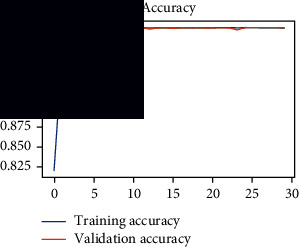	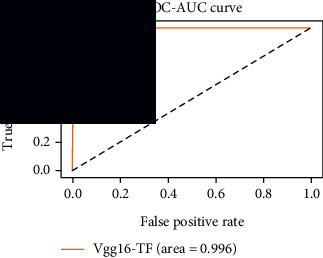	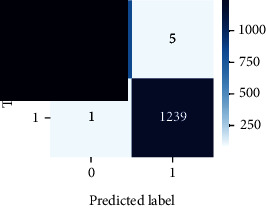

## Data Availability

The data used to support the findings of this study are included within the article.
